# The Force of Numbers: Investigating Manual Signatures of Embodied Number Processing

**DOI:** 10.3389/fnhum.2020.590508

**Published:** 2021-01-11

**Authors:** Alex Miklashevsky, Oliver Lindemann, Martin H. Fischer

**Affiliations:** ^1^Potsdam Embodied Cognition Group, Cognitive Sciences, University of Potsdam, Potsdam, Germany; ^2^Department of Psychology, Education and Child Studies, School of Social and Behavioural Sciences, Erasmus University Rotterdam, Rotterdam, Netherlands

**Keywords:** ATOM, embodied cognition, finger counting, grip force, mental number line, number processing, numerical cognition

## Abstract

The study has two objectives: (1) to introduce grip force recording as a new technique for studying embodied numerical processing; and (2) to demonstrate how three competing accounts of numerical magnitude representation can be tested by using this new technique: the Mental Number Line (MNL), A Theory of Magnitude (ATOM) and Embodied Cognition (finger counting-based) account. While 26 healthy adults processed visually presented single digits in a go/no-go n-back paradigm, their passive holding forces for two small sensors were recorded in both hands. Spontaneous and unconscious grip force changes related to number magnitude occurred in the left hand already 100–140 ms after stimulus presentation and continued systematically. Our results support a two-step model of number processing where an initial stage is related to the automatic activation of all stimulus properties whereas a later stage consists of deeper conscious processing of the stimulus. This interpretation generalizes previous work with linguistic stimuli and elaborates the timeline of embodied cognition. We hope that the use of grip force recording will advance the field of numerical cognition research.

## Introduction

People use numbers to represent magnitudes, operate, and communicate quantitative information (Dehaene, 1992; Núñez, 2017). The nature of our mental representations of magnitudes is currently under debate. Several hypotheses have been proposed and we evaluate three prominent accounts here, focusing on the magnitude meaning of number symbols. One hypothesis about magnitude knowledge is A Theory of Magnitude (ATOM), which suggests a shared cortical mechanism for time, space, and all other quantities (Walsh, 2003, 2015). Another hypothesis is a Mental Number Line (MNL), suggesting grounding of magnitude knowledge in spatial experience with smaller numbers being represented on the left side and larger numbers on the right side of a magnitude-ordered number sequence in our mind. A third hypothesis, derived from the Embodied Cognition view, assumes that habitual sensory and motor experiences such as finger counting become part of the cognitive representation of numbers from 1 to 10. In the following section, we review the evidence for each of these three hypotheses about the cognitive representation of number knowledge in more detail to motivate specific predictions for performance in our study and then demonstrate how a novel technique—grip force recording—can be used for testing each of those theoretical accounts.

### A Theory of Magnitude (ATOM)

ATOM predicts a “monotonic mapping of quantities: bigger, faster, brighter, further in one domain should correlate with bigger, faster, brighter, further in another” (Walsh, 2015, p. 557). These dimensions, where the perceived increase is associated with qualitatively larger excitation in the same physiological system (e.g., stronger stimulation of photoreceptor cells on the retina) are called prothetic (Stevens, 1957; Lindemann and Fischer, 2015). In contrast, metathetic sensory dimensions are characterized by a substitution of the activity pattern. For example, a high compared to a low pitch does not result in a more intensive stimulation of sensory cells but comprises stimulation of a different part of the cochlea. Following ATOM, prothetic dimensions can be naturally mapped onto each other, since they are based on the same physiological mechanisms.

There is considerable support for ATOM. First, consider the distance effect—easier discrimination of two numerical values that are farther apart. This effect was found for numerical symbols as well as for other quantitative domains, such as physical sizes, line lengths, or luminance levels (see Cohen Kadosh et al., 2008, for review). This suggests that, when comparing the meaning of two abstract symbols, we rely on the same mechanism that is also used for comparing the perceptual properties of two real objects. Next, consider the size congruity effect (Henik and Tzelgov, 1982) where the physical size of a number symbol influences the speed of its magnitude classification, and the magnitude meaning influences, in turn, the speed of its classification by physical size.

Also consistent with ATOM, task-irrelevant numbers interact with grip aperture in a lifting task (Andres et al., 2004, 2008; Lindemann et al., 2007; Namdar et al., 2014). Specifically, larger numbers facilitate wider (or power) grip apertures and smaller numbers facilitate narrower (or precision) grip apertures. This link is bidirectional: perceiving graspable small vs. large objects also affects the processing of small vs. large numbers differently (Ranzini et al., 2011). Further supporting evidence for ATOM comes from the FoNA effect—Force Numerical Association of response codes, the association between response force and numerical magnitude: participants respond faster to small numbers with weak responses and large numbers with forceful responses (Vierck and Kiesel, 2010; Krause et al., 2013). However, no direct automatic association between numerical magnitude and the amount of applied force was found (Fischer and Miller, 2008; Vierck and Kiesel, 2010), as one would expect: the mapping between force and numbers is categorical, i.e., large numbers are associated with forceful responses, but relatively larger numbers do not lead to relatively stronger responses. This raises the question if there are Force-Numerical Associations without response codes (see also in the “Mental Number Line (MNL)” section). A paradigm without an explicit motor response would clarify this theoretically important issue.

### Mental Number Line (MNL)

The MNL hypothesis suggests a spatially oriented mental representation of numbers with smaller numbers on the left and larger numbers on the right side. Most of the evidence for MNL comes from studies of spatial-numerical association (SNA) of response codes (Dehaene et al., 1993)—it is easier for participants to respond to small numbers when the response is left-lateralized, and to large numbers when the response is right-lateralized. SNA effects are not effector-specific and can be found with left-right hand button press responses, finger movements (Fischer, 2003), eye movements (Myachykov et al., 2016), foot responses (Schwarz and Müller, 2006) as well as full-body movements (for reviews see Fischer and Shaki, 2014; Toomarian and Hubbard, 2018). This ubiquity of spatial-numerical associations suggests that SNA is related to the left vs. right side of peri-personal space (location hypothesis) and not to the left vs. right hand (effector hypothesis). Studies with crossed hands support the location hypothesis (Dehaene et al., 1993, Experiment 6). The MNL is an attractive hypothesis because it accounts even for negative numbers that do not have distinct sensory or motor experiences (Mende et al., 2018) and for arithmetic operations as movements along the MNL (Pinhas and Fischer, 2008).

An influential theoretical account of SNA effects is the dual-route model of Gevers et al. (2006b). It suggests that numerical processing involves two routes: a conditional route, or mapping of motor responses, is created by the task instruction (e.g., odd numbers—left response; even numbers—right response); and an unconditional route, involves automatic activation of magnitude information and its associations with space. According to this model, SNA effects can only occur when there is a lateralized response, as the effect reflects interactions between magnitude information and response selection (Gevers et al., 2006a). The dual-route model explains the fact that SNA is stronger in slower responses (see Wood et al., 2008, for a meta-analysis) and accurately predicts the optimal conditions for detecting SNA: the earlier the number semantics is activated and the longer it takes to perform a response, the stronger the resulting SNA effect should be (see Pressigout et al., 2019, for experimental support).

However, several recent studies demonstrated SNA without any spatially selective response. For example, Myachykov et al. (2016) presented participants with auditory English numbers and found SNA-congruent spontaneous ocular drift on a blank screen already 450 ms after auditory number onset. Similarly, SNA was found in eye movements during counting (Hartmann et al., 2016; Holmes et al., 2016). These results challenge the dual-route model, as no SNA should be expected in tasks without explicit responses. Enforcing this idea, Shaki and Fischer (2018b) used an implicit association test without lateralized responses to demonstrate the absence of horizontal SNA in the parity judgment task; yet, with the same paradigm, they found horizontal SNA in a magnitude comparison task where number meaning is explicitly activated. This task-dependence of findings indicates a strong need for new paradigms to investigate the automatic nature of SNA and their functional role in number understanding without lateralized responses or, even better, without any explicit motor response at all.

### Embodied Cognition

Embodied cognition claims that sensory and motor activation present during concept acquisition remains associated with conceptual knowledge (Barsalou, 2008; Fischer, 2012; Fischer and Coello, 2016). This can explain why adults who learned to start counting on their right hand show a diluted or even reversed SNA effect (Fischer, 2008); they have learned to associate small numbers with right instead of left space. Several studies found links between finger counting habits and number processing both when actively responding with fingers (Di Luca et al., 2006) and when passively receiving finger stimulations (Sixtus et al., 2020). Finger gnosia, the ability to distinguish between one’s fingers in both sensory and motor discrimination tasks, predicts mathematical performance in children (Fayol et al., 1998; Costa et al., 2011), although the exact functional role of finger counting in mathematical cognition is debated (Fischer et al., 2012). While fingers are an almost universal mechanism for number learning in children, the exact patterns of finger counting exhibit cross-cultural variability: people in Western cultures tend to start counting with their left thumb, while in Eastern cultures the majority of people starts with the right pinky^1^ (Lindemann et al., 2011). Finger counting associations with numbers might have a non-spatial motoric nature: adopting the posture facilitates the processing of a corresponding digit, while merely observing such a posture does not have this effect (Sixtus et al., 2017). Moreover, finger counting habits modulate activity in the premotor cortex in response to small numbers, indicating the importance of motor planning for finger-digit associations (Tschentscher et al., 2012).

### Interactions Between Representational Mechanisms

Despite a relatively large body of literature on the three different approaches reviewed above, little is known about interactions between the mechanisms postulated by the ATOM, MNL, and embodied cognition hypotheses for magnitude representation; yet such knowledge is necessary to build a comprehensive model of number processing which integrates non-symbolic and symbolic components (see Núñez, 2017). Only a few studies have directly compared different accounts. For instance, Wiemers et al. (2017) measured space- and size-congruity effects within one experiment, thus simultaneously testing the MNL and ATOM accounts. They presented participants with numerically large and small numbers written in physically large or small fonts either on the right or on the left side of the screen. The authors analyzed a congruency sequence effect, or Gratton effect (Gratton et al., 1992) in response times for these two features—the numerical and the physical size. Gratton effect consists of faster conflict solving in incompatible trials if they were preceded by other incompatible trials, but only if this incompatibility was in the same dimension. Thus, the Gratton effect allows to distinguish between qualitatively different features of a stimulus processed by different cognitive means. The authors found two Gratton effects independent from each other—one for size dimension and another for space dimension (horizontal location). The authors interpreted these results as evidence of intra-individual parallel processing of both ordinal (position on the MNL, spatial information) and cardinal (magnitude, size information) aspects of number. Both effects were present equally strongly in early and late reaction times, which indicates that these two numerical aspects are processed simultaneously.

In contrast to this view, Krause et al. (2013) interpreted FoNA and SNA effects as signatures of alternative mapping strategies for mapping magnitudes either onto force (FoNA) or onto space (SNA), with different strategic preferences across participants. They found a correlation between gray matter volume in the left angular gyrus and strength of individual FoNA effects, as well as a correlation between gray matter volume around the right precuneus and strength of individual SNA effects. Importantly, there was no significant correlation between individual SNA and FoNA effects. Thus, according to these authors, different individuals rely more on either spatial or non-spatial representations of magnitude even when they perform the same task (parity judgment).

A few studies contrasted MNL and embodied accounts of magnitude processing. Fischer (2008) reported a typical SNA amongst left-starters while right-starters demonstrated no reliable SNA as a group and some individuals even showed a reverse mapping. The lack of a fully reversed SNA in right-starters suggests a within-individual coexistence of both MNL and finger-counting based magnitude representations, which might then have additive effects. This result was, however, not replicated by Tschentscher et al. (2012) who found similar SNA in both left- and right-starters, and by Fabbri (2013) who found even stronger SNA in Italian right-starters. At the same time, Tschentscher et al. (2012) revealed modulation of lateralized brain activity by finger counting habits, and no correlation between the strength of this modulation and individual SNA, again confirming independence of finger-based and MNL-based representations.

Di Luca et al. (2006) found that finger counting habits predict performance in number processing better than the MNL-based mapping in Italian participants (who were all right-starters), irrespective of whether the hand was in the palm-up or palm-down position. However, Brozzoli et al. (2008) stimulated thumb or pinky in the palm-up and palm-down positions and found that small numbers prime left-positioned fingers while large numbers prime right-positioned fingers regardless of finger-counting associations, i.e., they found a prevalence of MNL in all conditions. Riello and Rusconi (2011) asked participants to respond to numerical stimuli with index and middle fingers in palm-up or palm-down postures and found that only one hand demonstrated the SNARC affect—the hand whose position currently matched finger counting habits with the MNL. The latter finding thus suggests a co-existence of both reference frames and their summation. Note, however, that while Di Luca et al. (2006) and Riello and Rusconi (2011) asked their participants to respond with fingers, i.e., perform a hand motor action, Brozzoli et al.’s (2008) participants’ task was to detect stimulation of fingers and then to give responses with a foot pedal, i.e., no hand action was involved. This might make these studies non-comparable: perhaps active hand movement is a prerequisite for finding finger-number associations that are grounded in the motor system while mere detection tasks put more emphasis on space and thus pre-activate the MNL?

### The Present Study

The goal of the present study was to examine spontaneous grip force fluctuations to study the continuous activations of magnitude concepts in an attempt to clarify the contributions of ATOM, MNL, and embodied representations of number knowledge. Grip force sensors were previously used to measure spontaneous motor activity during language processing (Frak et al., 2010; Aravena et al., 2012, 2014) or action observation (Blampain et al., 2018). They are now an established tool to track the dynamics of cognition with high temporal resolution (for review and methodological details, see Nazir et al., 2017). For example, a spontaneous increase in grip force accompanied listening to action descriptions like “Fiona lifts the dumbbells,” while no such increase was observed for abstract phrases, such as “Edmonde loves the flower bush in her garden.”

Interestingly, grip force is modulated by purely linguistic parameters, such as negation (Aravena et al., 2012) or modality of the action (Aravena et al., 2014), thus signaling that it reflects semantic processing. During bimanual force recording, both hands exhibit activity in response to motor-related linguistic stimuli (da Silva et al., 2018). A bimanual approach also addresses the possibility of a compensatory relationship between the two brain hemispheres in any given task. The present study extends the bimanual grip force method to the domain of number processing which has never been done before to the best of our knowledge. Importantly, the used equipment (see below) enabled us to record grip force continuously with a temporal resolution of one ms and thus allowed an exploration of the cognitive processes during number comprehension online. This is advantageous compared to reaction time registration which only provides the resulting number, a sum of multiple processes, including response-related ones, as discussed above.

**Figure 1:**
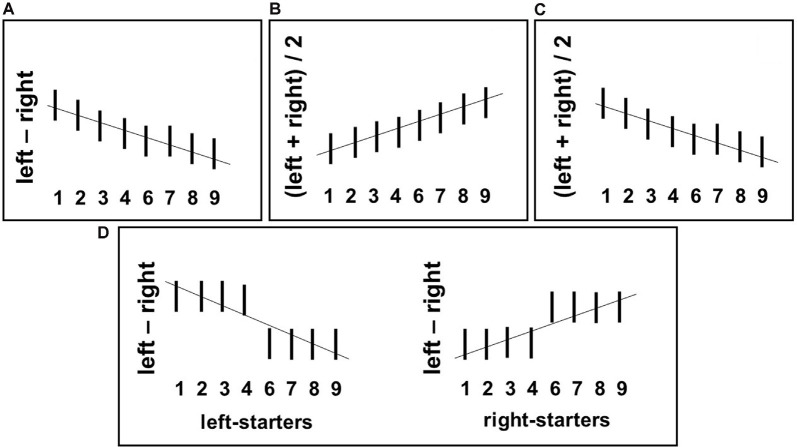
Predicted relationships between numerical stimuli (horizontal axes) and the grip force measures calculated by the formulae given next to the vertical axis in each plot. **(A)** Mental number line (MNL)-based prediction: gradual decrease of differential force dForce (i.e., left minus right-hand force) with an increasing number magnitude. **(B)** FoNA-based prediction: gradual increase of Average Bimanual Force (ABF) [(left + right-hand force)/2], with an increasing number magnitude. **(C)** Association between smaller numbers and precision grip used to hold the force sensors (Andres et al., 2004): gradual decrease of ABF with increasing number magnitude. **(D)** Embodied Cognition-based prediction: interaction between number magnitude and counting preferences, namely categorical association of small numbers with the left hand and large numbers with the right hand in left-starters leading to drop of dForce after number 4; the opposite pattern in right-starters.

Based on the recent hypotheses for number representation reviewed above, we derived the following five specific predictions for our study and visualized them in Figure 1.

*Hypothesis 1*: If a spatially oriented MNL is activated during number processing, there should be an increase of force in the right hand in response to large numbers (6–9) and in the left hand in response to small numbers (1–4); this trade-off between hands should be continuous^2^, see Figure 1A.

*Hypothesis 2*: Numerical magnitude affects the overall applied grip force. If a general magnitude is activated, then gradually increasing force in response to larger numbers should be found in both hands; this effect should be continuous, see Figure 1B. Alternatively, ATOM makes an opposite prediction: there might be a mapping between the numerical magnitude and grip aperture (as in Lindemann et al., 2007). In this case, larger numbers should lead to a wider grip and, accordingly, to weaker force, also in both hands. Again, this effect should be continuous, see Figure 1C.

*Hypothesis 3*: Finger counting preferences modulate the linkage between number size and grip force. Based on an embodied cognition account, grip force might increase for small numbers (1–4) in the hand participants use to start counting on their fingers; the opposite should be true for larger numbers (6–9) and the other hand. This effect should be categorical, as only one sensor for each hand is used in our study, see Figure 1D.

*Hypothesis 4*: If any of the described representations (MNL, ATOM, or finger-based representations) will be activated in parallel and reflect different aspects of number semantics (intra-individual variability), they should not interact and their effects must be additive (as in Wiemers et al., 2017).

*Hypothesis 5*: Individuals have a preference to map numbers either to space or to force. If there are individual differences in utilizing different mechanisms (inter-individual variability, as suggested by Krause et al., 2013), for some participants FoNA effect should be found, whereas for others SNA. At the individual level, a negative relationship between FoNA and SNA can be expected because participants who rely on one kind of representation do not need another one.

### Timing of Number Processing

Examining number processing with our proposed method of continuous force recording calls for* a priori* specification of the temporal characteristics of the expected effects. So far, few experiments examined explicitly the timing of number processing. However, previous work using electroencephalography (EEG) is informative. Specifically, Hsu and Szücs (2012) used a neural adaptation paradigm and found evidence for two stages of magnitude processing in the EEG signal: an earlier process emerging at around 220–260 ms (left parieto-occipital sites), reflecting initial stages of magnitude analysis; and a later process at around 420–450 ms (central sites), reflecting higher-level categorical processing of numbers, or N400-like effect. Gut et al. (2012) found effects of numerical magnitude in the first negative-going component of the EEG, the so-called N1 amplitude (120–250 ms). Gevins and Cutillo (1993) also related the N1 to initial stimulus processing. Myachykov et al. (2016, Experiment 1) found a significant effect of numerical magnitude on spontaneous eye-movements in a no-go task within 450–600 ms with the auditory presentation of numbers^3^.

Pulvermüller (2012) identified three features of the comprehension process: immediacy (early onset of signatures of sematic processing), automaticity, and functional relevance. As a consequence, if motoric or spatial effects reflect an understanding of number concepts, these effects must appear early and automatically. However, most previous studies used reaction time as a dependent measure, which does not identify the critical time window when SNA or FoNA effects first appear (see Balota et al., 1992). Recent eye- or mouse-tracking studies investigate the timing of single or multi-digit number processing and arithmetic processes (for reviews, see Fischer and Hartmann, 2014; Faulkenberry et al., 2018). Grip force recording provides us with further insights into the temporal dynamics of number processing. Thus, a hypothesis about the timing of the effects can be formulated.

*Hypothesis 6*: According to the results of Hsu and Szücs (2012) we expect to find two time-windows reflecting two stages of number processing—an earlier (220–260 ms) and a later (420–450 ms) one.

## Materials and Methods

### Participants

Twenty-six psychology and linguistics students at the University of Potsdam participated in the study for course credit (two males, mean age 25 years, range 18–46 years). All participants reported normal or corrected-to-normal vision and absence of either motor diseases or movement-affecting medications. All but two participants (8% of the sample) were right-handed by self-report. All participants signed an informed consent form. The study was approved by the local Ethics Committee (study number 75/2016).

### Equipment and Data Acquisition

**Figure 2:**
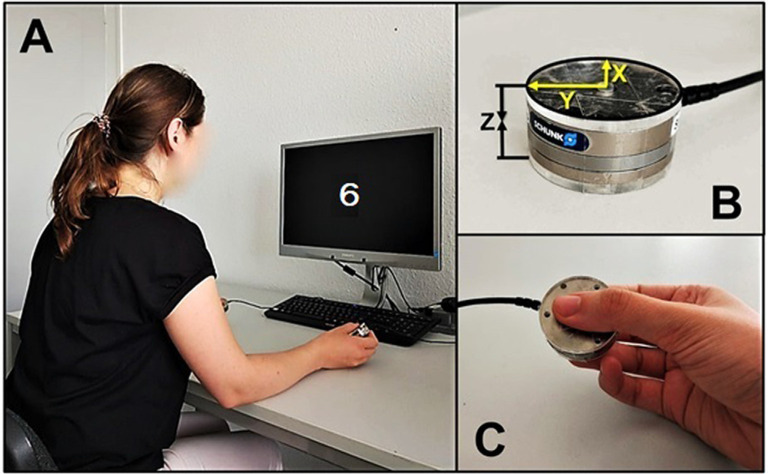
**(A)** Bimanual force recording setup. **(B)** Grip force sensor (X—longitudinal, Y—radial, Z—compression forces). **(C)** The way participants held the sensors (around 45° relative to the table surface, not strictly controlled).

Our method followed closely the one recommended by Nazir et al. (2017) for single-sensor recording. Both sensors were stand-alone load cells manufactured by ATI Industrial Automation, USA (www.ati-ia.com/Products/ft/sensors.aspx). They resembled large metal coins with 40 mm diameter and 14 mm height and each weighed 57 g. We used neither large plastic covers as Nazir et al. (2017) nor additional weights, as da Silva et al. (2018). Instead, each sensor was covered from both contact sides with a 3 mm plastic cover of the same diameter as the sensor itself (40 mm), resulting in a total thickness of 20 mm and a total weight of 65 g per sensor (see Figure 2). Our sensors record force dynamics with ms resolution along three orthogonal axes but only Fz force along the vertical axis through the sensors was analyzed and is reported here. Two PCs were used: one for running the experiment under OpenSesame software (Mathôt et al., 2012), and the other one for force data acquisition under Experiment software (Krause and Lindemann, 2014). The first PC sent a trigger at the beginning of each trial; this trigger was later used to identify a corresponding time point in the force data file.

### Task, Stimuli, and Procedure

Participants sat at a desk and held one sensor in each hand at an angle of around 45° relative to the table surface with the thumb on one side and both index and middle finger on the other side. Participants’ elbows rested on the table while their hands held the sensors, thus preventing sensor slippage (see Figure 2). The distance between sensors varied from 30 to 50 cm and was not strictly controlled but both were equidistant from each participant’s mid-sagittal plane.

Before data collection, participants were trained to apply a holding force ranging between 1.5 N and 3 N with each hand. The sensors were represented on the screen as two circles which changed their color from green (“too weak”) to red (“too strong”) with the acceptable force range indicated by gray color. As soon as participants managed to turn both circles into gray, they were asked to keep the force at this level during the whole testing session. After participants held the sensor with the required force for three seconds without crossing these thresholds, data collection started automatically. This calibration procedure was later repeated after each break.

We collected data in the no-go trials of a 1-back go/no-go task. This task ensures active processing of digits while at the same time removing any explicit responses to stimuli of interest (Chen et al., 2008). This means that grip force recordings are not contaminated by overt responses, which typically generate large artifacts in these recordings. Participants monitored a stream of digits presented centrally on a screen, said “yes” whenever the digit repeated the previously presented one, and otherwise stayed silent. Note that participants were explicitly instructed to hold the sensors always with the same force and only respond to stimuli verbally, i.e., no motor response was required and all force oscillations observed in the experiment were completely unconscious and unintentional. Importantly, the no-go trials required neither explicit semantic processing nor motor or verbal responding. Any magnitude-related effects will therefore satisfy the requirement of automaticity (Pulvermüller, 2012).

Stimuli were the digits from 1 to 9 excluding 5. The size of digits was 0.7 cm and the distance from participants’ eyes about 60 cm, which gives 0.67 degrees of visual angle^4^. Digits were presented pseudo-randomly in mini-blocks of four successive digits so that small digits were intermixed with other small digits (e.g., 4, 1, 3, 2) and large digits with other large digits (e.g., 6, 8, 7, 9). These magnitude-defined blocks alternated (e.g., small-large—small-large, etc.). Remember that the task was to compare the currently presented number with the previous one (1-back task, see above). The alternation of mini-blocks ensured that small numbers were almost always compared with small numbers and large numbers with large numbers.

**Figure 3:**
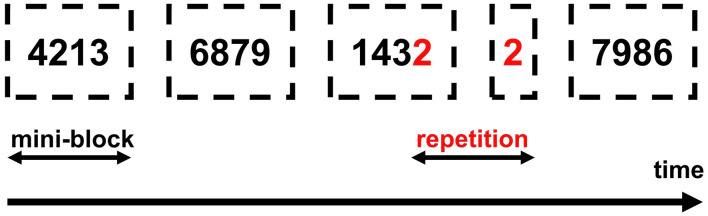
Experimental procedure. Dashed lines depict mini-blocks of small/large numbers. Participants could see only one (black) number per trial and were not informed of the mini-block structure.

Digit repetitions (go-trials) occurred always between mini-blocks (see Figure 3). Once the participant detected a repetition and said “yes” the experimenter recorded the response by clicking a mouse button. The mouse was connected to the presentation PC and all responses were recorded through OpenSesame. Participants received two types of feedback: a red frowny for false alarms and a sandglass icon if no response to a go-stimulus was delivered within 2,000 ms. Each number was presented for 2,000 ms and the inter-trial-interval was 500 ms. Numbers were presented in two different fonts and with a randomly selected rotation angle from −45 to 45 degrees, to exclude simple visual identification of repetitions and to facilitate active number recognition.

### Design

We used a mixed factorial design: 2 (Hand: left/right, within-participant factor) × 2 (Number: small/large, within-participant factor) × 2 (Counting Preference: left-starter/right-starter, between-participant factor). In some analyses for a more detailed picture, we considered Number as a factor with eight levels (1–9, without 5).

Each number was presented 92 times, thus resulting in 736 trials overall plus 20 trials of practice. The experiment lasted for around 40 min. Every 7–10 min participants had a break until they decided to continue, but no less than 2 min (time necessary for the recording PC to acquire the force data).

### Questionnaires

After the experimental session, participants answered a questionnaire about their sex, age, mother tongue, foreign language(s), field of study, handedness, vision or motor problems, and musical instrument skills^5^. Next, participants underwent a finger counting test. First, the experimenter asked participants to rotate their chairs and to take a symmetrical and seated position in front of the equally seated experimenter. Then participants were told to shake both hands in the air. After that, participants were asked to count ostentatiously from 1 to 10 by using their fingers. The experimenter kept a symmetrical posture during testing and only used verbal instructions, never demonstrating to participants how they should count. This ensured the retrieval of as natural a counting pattern as possible by removing all spatial and motor interferences, imitation strategies, and the impact of lateralized communicative cues. After the participant had counted to 10 the experimenter marked the pattern of counting in a separate questionnaire, including starting hand, starting finger, and whether the palms were closing or opening during the counting.

### Data Analysis and Results

From the 26 participants, 22 (85%) reported German as their native language; other native languages were Russian, English, Chinese, and Turkish. Fourteen participants (54%) did not play any musical instruments, the others played guitar, piano, flute, or other instruments. Thirteen participants (50%) started counting with their left hand, the other 13 (50%) with their right hand. Twenty-three participants (88%) started counting with their thumb, whereas two participants (8%) started with the pinky and one (4%) with the index finger. All but one participant (96%) closed their palms during counting. No association between playing a musical instrument (playing vs. not playing) and starting hand (left- vs. right-starters) was found ($\chi_{\left(1,N=26\right)}^{2}$ = 0, *p* = 1). For every participant, the following averaged force metrics were calculated separately for left and right hand: mean force, the standard deviation of force, maximum force per trial, and skewness of force distribution. Additionally, the mean correlation of force between both hands was calculated for each participant and Fisher *z*-transformation^6^ was applied to correlation coefficients to normalize their distribution. Each of these variables was then submitted to a one-way ANOVA with either musical instrument (yes/no) or counting preference (left/right) as a predictor. Since no differences were found in any of the force metrics, all data were collapsed and submitted to further analyses.

#### Data Preprocessing

Trials with false alarms (i.e., no-go trials where participants responded) and missing responses (go trials where participants did not respond) were discarded from the analysis. Those trials were generally infrequent but one participant was completely excluded because of relatively low accuracy (89%), the other 25 all had accuracy >97% (mean = 99%) and only their data were further analyzed.

The preprocessing of grip force data followed the recommendations of (Nazir et al., 2017, Experiment 2). Before analysis, data were filtered at 15 Hz with a fourth-order, zero-phase, low-pass Butterworth filter. Single epochs were extracted from the vertical Fz signal, starting 200 ms before and ending 1,000 ms after stimulus onset. The global drift in force across the experiment was corrected by subtracting the average force from 20 ms intervals before stimulus onset from each ensuing epoch. As a result, grip force always crosses the zero point at the start of each trial and negative force values reflect a vertical grip force less than that at the moment of stimulus presentation, not the absence of force. Similar to Nazir et al.’s (2017) procedure, maximum and minimum thresholds were applied (± 500 mN^7^) to remove movement artifacts and identify participants with unacceptably large force variability. The proportion of trials where force exceeded one of the thresholds varied across participants from 0 to 15% (mean = 3%) and although such trials were discarded no participant was excluded because of this criterion.

**Figure 4:**
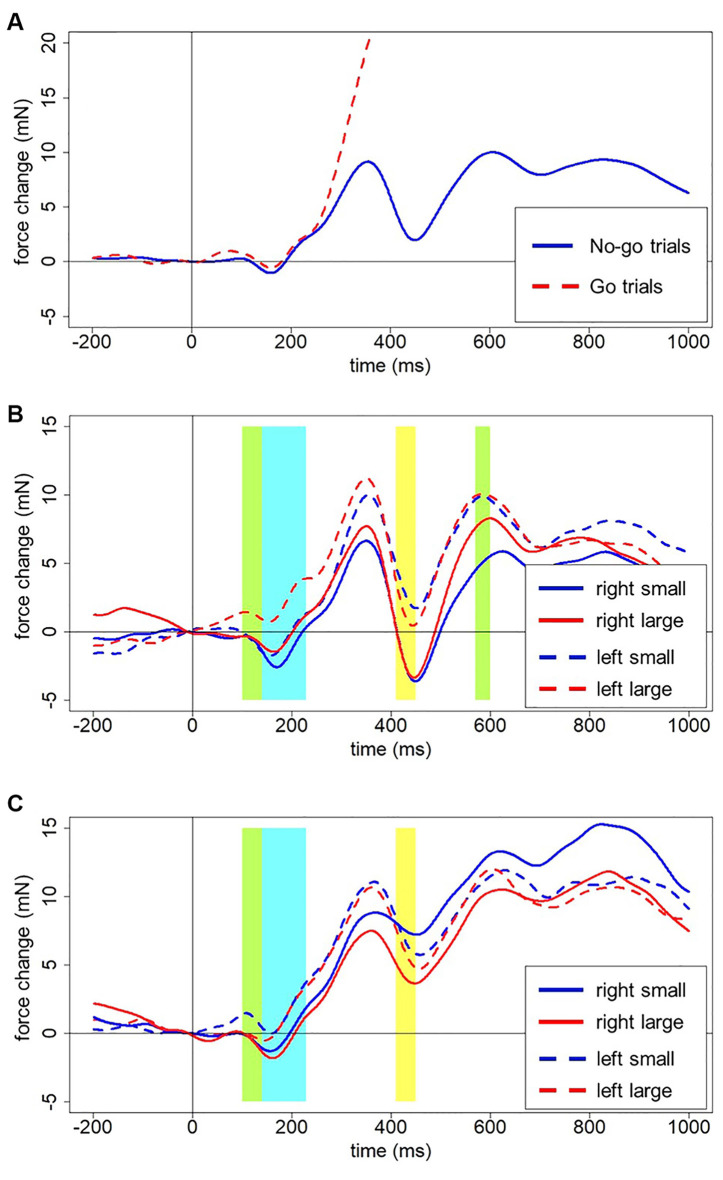
**(A)** Averaged force profiles across all go and no-go trials of all participants. Forces for go and no-go trials clearly diverge around 250–300 ms after stimulus presentation. **(B)** Force profiles for left-starters (no-go trials). Green areas indicate an interaction between Hand, Number, and Counting Preference. The Blue area indicates the main effect of the Hand. The yellow area indicates the main effect of Number (see description in the text below for details). **(C)** Force profiles for right-starters (no-go trials). The green area indicates the interaction between Hand, Number, and Counting Preference. The Blue area indicates the main effect of the Hand. The yellow area indicates the main effect of Number (see description in the text below for details).

The outcomes are depicted in Figure 4. Figure 4A shows the multi-phasic nature of grip force changes across time after digit onset, averaged across all accepted trials. In go-trials, the force increases 250–300 ms after stimulus onset to about 90 mN by the end of the epoch, clearly diverging from the force in no-go trials. In no-go trials, we see a slight increase and immediate decrease of force already before 200 ms after stimulus onset, followed by a first large peak at around 350 ms and a sharp dip at around 450 ms. Another peak of about the same amplitude is reached at 600 ms with a small decrease following. The third and most gradual increase reaches its peak at 850 ms and then grip force gradually declines. In close analogy to neurophysiological EEG signals from the scalp surface, these spontaneous force fluctuations in the hands reflect underlying cognitive processes that will be discussed in more detail below.

These patterns generally do not differ for the two hands, or small and large numbers plotted separately, neither between participants nor for left- vs. right-starters studied separately. Left-starters demonstrate a steeper drop after 400 ms, whereas right-starters demonstrate a higher peak after 700 ms (compare Figures 4B,C).

#### Data Analysis: Hand, Number and Counting Preference at the Group Level

To explore the force data according to the hypotheses formulated above, we aggregated forces by Number (small/large) and Hand (left/right) within participants, and then between participants for left- and right-starters separately. These data were submitted to a cluster permutation analysis (R Core Team, 2017, package “permuco”; Frossard and Renaud, 2018) with Number and Hand as within—and Starting Preference as between-variables. Five thousand permutations were performed and TFCE (Threshold-Free Cluster Enhancement, see Ehinger, 2019, for more details) correction for multiple comparisons was used.

Cluster permutation analysis is a bootstrapping method used for the processing of continuous data. In this analysis, the labels of different conditions are shuffled, thus creating a random structure of the data. After each permutation, the analysis is performed according to newly assigned labels, a *t*-statistics is calculated and the mass of the clusters exceeding a significance threshold is stored. Multiple repetitions of this procedure result in an approximation of the distribution of cluster-masses given a random data structure. Finally, the comparison between this bootstrapped cluster-masses and the actual cluster-mass provides an estimation of the likelihood that the observed result is due to the experimental design (see also Maris and Oostenveld, 2007, for more details). Our analysis indicated several time windows with results approaching the significance threshold. Particularly, the effect of hand was most pronounced within 140–230 ms after stimulus onset, and, most importantly, there was an interaction between all three factors (Hand, Number, Counting Preference) within 100–140 ms and 570–600 ms after stimulus onset. Force data were then aggregated within the identified time windows and submitted to repeated-measures ANOVAs^8^ with Hand (left/right) and Number (small/large) as within-factors and Counting Preference (left/right-starters) as between-factor.

**Figure 5:**
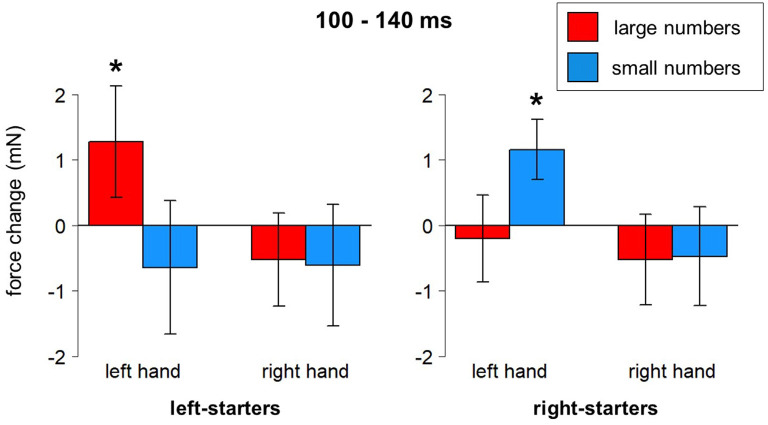
Number*Hand*Counting Habits interaction (100–140 ms after stimulus onset). Vertical bars represent standard errors. Stars represent conditions that are significantly different from most of or all other conditions (see main text for details).

Several clear results were obtained. In the time window from 140–230 ms after stimulus onset, there was a reliable effect of Hand on grip force. Specifically, the left hand showed larger force than the right hand during that time interval, *F*_(1,23)_ = 6.69, *p* = 0.016, partial eta-squared = 0.23; see blue frame in Figures 4B,C. A significant interaction between Hand, Number, and Counting Preference was found in the time window from 100 to 140 ms, *F*_(1,23)_ = 5.73, *p* = 0.025. A *post-hoc* LSD test revealed a significant increase of force in the left hand in response to large numbers in left-starters (*p*-values range from 0.01 to 0.007) and the opposite pattern in right-starters, i.e., increasing force in the left hand in response to small numbers (*p*-values ranging from 0.055–0.02), see Figure 5. The right hand did not demonstrate any differences in response to our experimental manipulations. This interaction is represented by the first green window in Figure 4B and the green window in Figure 4C.

**Figure 6:**
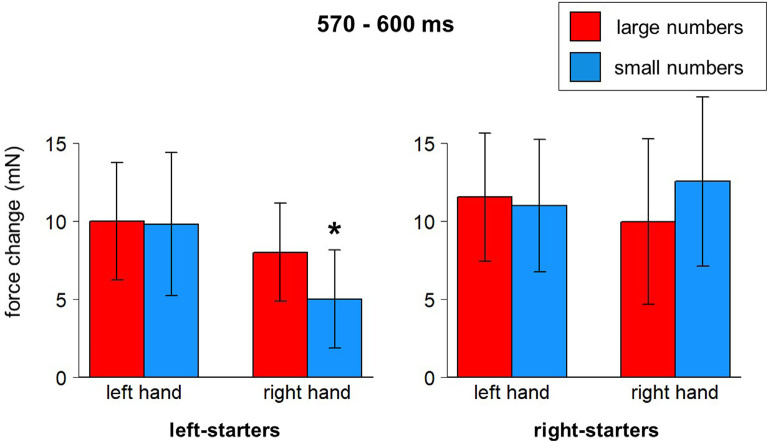
Number*Hand*Counting Habits interaction (570–600 ms after stimulus onset). Vertical bars represent standard errors. Stars represent conditions that are significantly different from most of or all other conditions (see main text for details).

A significant interaction between Hand, Number, and Counting Preference was found in the time window from 570–600 ms, *F*_(1,23)_ = 4.82, *p* = 0.039. The *post-hoc* LSD test demonstrated in the group of left-starters significantly lower force in the right hand in response to small numbers compared to other conditions (*p*-values from 0.033 to 0.001). In the group of right-starters no differences between conditions were found, although the right-hand force was marginally larger in response to small numbers than to large numbers, *p* = 0.075 (see Figure 6; also yellow windows in Figures 4B,C).

#### Data Analysis: Continuous Effects of Number on the Force

**Figure 7:**
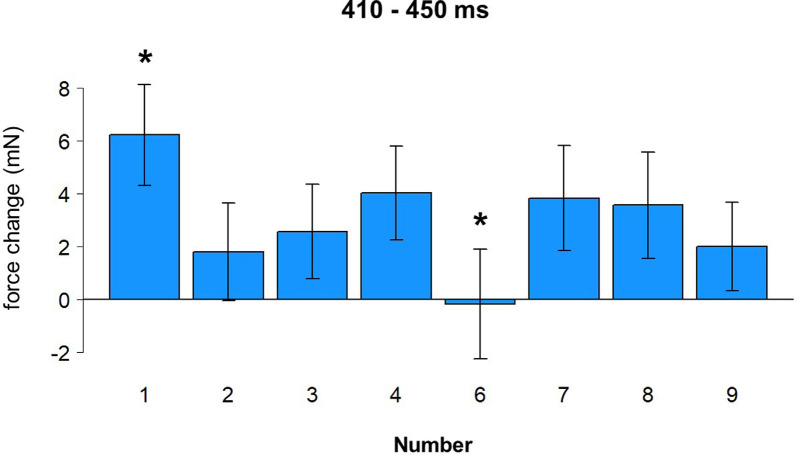
Effect of number (410–450 ms). Vertical bars represent standard errors. Stars represent conditions that are significantly different from most of or all other conditions (see main text for details).

To investigate the force changes as a function of the number, a more detailed analysis was performed with eight levels of the factor Number (1/2/3/4/6/7/8/9). With this only change, the same parameters were used for a cluster permutation analysis. As before, the analysis suggests an early time window from 150 to 230 ms after stimulus onset (Hand effect) and a later time window from 410 to 450 ms after stimulus onset (Number effect), although none of those reached significance in cluster permutation results. All data were averaged in the time window 410 to 450 ms after stimulus onset and submitted to repeated measures ANOVA with Number (eight levels) as a within-factor. There was a main effect of Number, *F*_(7,168)_ = 2.34, *p* = 0.027. A *post-hoc* LSD test revealed a larger force in response to number 1 compared to numbers 2, 3, 6, and 9, all *p*-values < 0.03, and weaker force in response to number 6 compared to numbers 1, 4, 7, and 8, all *p*-values < 0.03. These results are shown in Figure 7 (see also yellow time window in Figures 4B,C).

#### Data Analysis: Direct Comparison of MNL and ATOM Accounts at the Individual Level^9^

**Figure 8:**
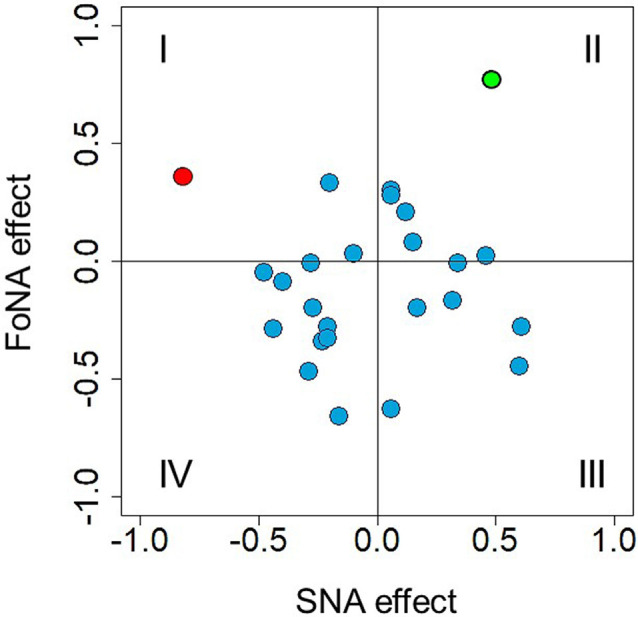
Individual Spatial-Numerical Association (SNA) effects plotted against individual FoNA effects. Quadrants I and IV represent traditional SNA, II, and III—reversed SNA. Quadrants I and II represent FoNA (as in Krause et al., 2013), III and IV—reversed FoNA (grip aperture effect, see Andres et al., 2004). The red dot represents a beta-coefficient of an SNA regression that reached significance (*p* = 0.01, quadrant I); the green dot represents a beta-coefficient of a FoNA regression that reached significance (*p* = 0.02, quadrant II).

Previous analyses revealed a time window of 410–450 ms after stimulus onset to be sensitive to number magnitude. This time window was selected to directly test MNL and ATOM accounts. Krause et al. (2013) identified independent brain structures responsible for SNA (right precuneus) and FoNA (left angular gyrus), respectively, and suggested that these two kinds of number representations are used by different groups of participants, i.e., they constitute inter-individual differences. Indeed, if there is an automatic mapping of numbers onto the MNL and this mapping is reflected in spatially congruent hand activation, then participants who rely on the spatial representation of numbers should demonstrate stronger force in the right hand in response to larger numbers and stronger force in the left hand in response to smaller numbers. Individual SNA, accordingly, were calculated for each participant and each number as a difference score: *dForce = left-hand force − right-hand force*, where positive values correspond to stronger association with the left side (hand) and negative values correspond to stronger association with the right side (hand). For each participant, individual regression slopes were extracted (regression coefficient analysis, see Pfister et al., 2013; also Fias, 1996) and then tested against zero with a one-sample *t*-test, to evaluate the strength of SNA. No overall difference from zero was identified, *t*_(24)_ = 0.139, *p* = 0.89. Standardized beta-coefficients for each participant as a measure of SNA effect size are presented in Figure 8 (where, per convention, quadrants I and IV correspond to traditional SNA and quadrants II and III to reversed SNA). Only in one participant (depicted in red), the regression reached significance (*p* = 0.01).

Possibly, some participants relied on ATOM-like representations, as Krause et al. (2013) suggested. These associations are not side/hand-specific and thus were calculated for each number for each participant as *ABF = (left-hand force + right-hand force)/2*, where larger values simply represent stronger force. Again, individual regression slopes were extracted for each participant and tested against zero by using a one-sample *t*-test, to evaluate the strength of FoNA. No difference from zero was found, *t*_(24)_ = −1.302, *p* = 0.21. Standardized beta-coefficients for each participant as a measure of FoNA effect size are presented in Figure 8 (y-axis), with reversed FoNA (grip aperture * force interaction) shown in quadrants III and IV, and direct FoNA (automatic force-magnitude mapping) shown in quadrants I and II. Again, only in one participant the regression reached significance (*p* = 0.02, represented in green on the plot).

Interestingly, when the only one participant with significant FoNA was removed from the sample, the rest of the sample did demonstrate significant reversed FoNA, *t*_(23)_ = −3.049, *p* = 0.006 (more data points with larger deviation from 0 on the y-axis in quadrants III and IV in Figure 8). This suggests that both representations predicted by ATOM might coexist in the sample and vary between individuals.

Finally, if SNA and FoNA indeed reflect qualitatively different representations used by different participants, there should be a negative relationship between them: if a participant relies on one type of representation, he or she will not use the other type. On the other hand, if these two kinds of representations reflect different aspects of numerical processing, namely ordinal and cardinal aspects, as Wiemers et al. (2017) suggest, then a positive relationship could be expected between the two measures at the individual level: both reflect number meaning and if the individual data reflect semantic processing at a higher or lower extent, all semantic effects should be more or less pronounced for a given individual. However, no reliably positive or negative correlation between individual SNA and FoNA was found, *r* = 0.053, *p* = 0.8.

## Discussion

In the present study, for the first time, bimanual grip force recording was used to investigate number processing. We contrasted three accounts of numerical magnitude representation: MNL, A Theory of Magnitude (ATOM), and embodied cognition. We will first discuss general patterns found in the grip force data and then summarize results according to our hypotheses.

First of all, the present study demonstrated that, independently of the numerical magnitude, the processing of all stimuli in our experiment caused systematic changes of grip force. For convenience, we will further refer to these changes as H (high force, peaks) and L (low force, dips) with a number representing a time point. For example, H350 means a peak with its highest point at around 350 ms, whereas L150 means a dip with its lowest point at 150 ms after the stimulus onset.

The task in the present study has two important aspects that should be considered when interpreting the results. First, it was a 1-back task, which implies two underlying cognitive processes: (1) matching a new item with a previous one; and (2) replacement of an old item with a newer one in memory (see Chen et al., 2008, for a detailed analysis). Second, it was a go/no-go paradigm, where only no-go stimuli were analyzed. This means that in all analyzed trials response inhibition should take place (see Gevins and Cutillo, 1993, for a similar task; see also Jonkman, 2006). In all no-go trials, irrespective of number magnitude, the same force pattern emerged:

• **H100** (100 ms after stimulus onset) followed by **L150**: we hypothesize that this signature in the grip force profile corresponds to the initial identification of the stimulus. There are no differences between go and no-go responses in this time window. However, a significant interaction between Hand, Number and Counting Preference (within 100–140 ms after stimulus onset) is observed: in the left hand, there is a significant force increase in response to large numbers in left-starters; the opposite is true for right-starters: a left-hand force increase in response to small numbers. Thus, initial processing even at this very early stage led to embodied effects of number semantics—numerical information interacts with individual finger counting experience, although in a rather unpredicted way (see below). The timing of this finding is generally in compliance with the findings of Hsu and Szücs (2012) and other results presented above, although the initial number processing stage comes in our data 100 ms earlier. This will be discussed below in more detail.
• **H250**: a slight deviation within a larger increase, where the averaged forces for go and no-go trials start diverging, forms an H250. This divergence of forces suggests that semantic processing has already happened and a decision to respond is being made in go trials. Right before it, in the time window 140–230 ms, the effect of hand appears with the left-hand force being stronger than the right-hand force.
• **H350** and** L450**: without any decrease after H250, the force profile goes up and reaches its peak at 350 ms, which we interpret as response inhibition^10^. The decrease of force is at the same time the end of a time window where the effect of number magnitude was significant (410–450 ms), with number 1 causing stronger force and number 6 weaker force than the other numbers. This time window might correspond to the second step of processing in the report by Hsu and Szücs (2012), or the effect found by Myachykov et al. (2016, Experiment 1). The exact effect of magnitude in our experiment is, however, not in compliance with any of the predictions (MNL, ATOM, or finger counting).
• **H600**, **L700**, and **H830** presumably reflect two further processes: replacement of an item in working memory (Chen et al., 2008) and anticipation of the next stimulus (Gevins and Cutillo, 1993). Again, a triple interaction between Hand, Number, and Counting Preference was found around H600, this time in the right hand: left-starters’ right-hand force significantly decreases in response to small numbers, whereas an opposite pattern was found for right-starters, whose right-hand force decreases in response to large numbers (though non-significantly, *p* = 0.075). This effect complies with an Embodied Cognition account, as the right hand is indeed associated with larger numbers in left-starters and with smaller numbers in right-starters. This effect demonstrates the role of embodied representations in the process of encoding the stimulus into working memory.

The results of the present study suggest that the grip force sensor does not simply register the level of activity of the motor system alone, but can also be sensitive to non-motor-specific cognitive processes of perception, memory, and decision making. Two stages of numerical processing, as suggested previously (Hsu and Szücs, 2012), might be thus reflected in the present data (which supports our *Hypothesis 6*). The initial stage of number processing can be compared to that of word processing (Pulvermüller et al., 2009), where all information, including semantics, is processed nearly simultaneously. In our data, however, this initial stage of processing appears 100 ms earlier than in the model by Hsu and Szücs, which might be the result of a mini-block structure—in 80% of trials numbers were followed by other numbers of similar magnitude—facilitating initial processing of similar stimuli (although this hypothesis contradicts the interference effect described later). Another source of differences in timing might be variations of task and procedure: in the study by Hsu and Szücs, participants’ task was to press a button in response to the color of stimuli in catch trials, and a random time interval of 2,400–3,600 ms was used between single trials. In our study, participants responded verbally, catch trials were repetitions of the n-1 stimulus, and the time interval between trials was kept constant (500 ms). Possibly, one or more of those differences allowed faster processing of stimuli in our study. It takes a visual stimulus 20–40 ms to reach the brain (Schlag and Schlag-Rey, 2002; Jain et al., 2015); another 18–20 ms are needed for the signal to come from the primary motor cortex to hand muscles (Rossini et al., 1999; Aravena et al., 2012) where it was measured by the sensors. After subtraction of these two numbers from 100 ms (the early effect described above), 40–60 ms remain for numerical processing.

The divergence of the go and no-go forces soon after this stage (H250) demonstrates that a decision is being made immediately after this point. The moment of decision making coincides with spontaneously increasing grip force in the left hand in all conditions (H350, n.s.), thus indicating some lateralized process associated with the right brain hemisphere. It cannot be excluded, however, that the right hand is generally better controlled, as most of the participants are right-handed; this could explain why similar effects in the right hand might be suppressed. To test this hypothesis, an experiment with handedness as a factor and a sufficient number of left-handed participants would be needed.

The unexpected direction of the effect in 100–140 ms (initial stimulus processing) should be considered together with the predicted direction of the effect in 570–600 ms (encoding of the stimulus in working memory for the next trial): According to the HANDLE model (García and Ibáñez, 2016), linguistic stimuli with motor semantics can lead to both facilitation and inhibition. The exact direction of the effect depends on the complexity of the linguistic stimulus, the complexity of the required motor response, and the timing between these two factors. In our case, the stimuli are not complex, but the *n*-back task requires participants to store every stimulus in working memory (H600), thus engaging neuronal resources for this stimulus. The block structure of our experiment leads to the fact that whenever a new stimulus is being processed (H100), a stimulus of similar magnitude (small or large) is already stored in working memory. Given that similar stimuli require similar neuronal resources, the resulting neural competition might induce the reversed effect.

Interestingly, the effect of finger counting preference at 100–140 ms was stronger for left-starters than for right-starters. This result is in agreement with one reported by Tschentscher et al. (2012), who also observed generally weaker motor cortical activation in right-starters in response to numerical stimuli compared to left-starters. To summarize, these results support our *Hypothesis 3* derived from an embodied cognition account (see “Introduction” section).

The MNL account predicts a link between the left side and small numbers and the right side and large numbers (see *Hypothesis 1* in the “Introduction” section). However, no automatic associations of numbers with a particular side/hand were found in the present experiment. The only interaction between number and hand, in the time window 100–140 ms, also included counting preference. Taking into account that Myachykov et al. (2016), Hartmann et al. (2016), and Holmes et al. (2016) did find SNA in eye movements in the absence of any motor responses, two possible explanations could be suggested: (1) the present study supports a dual-route account of the SNA effect, according to which the effect only occurs when there is an explicit lateralized response; or (2) the SNA effect is associated with space, not the effector, and it can be found in eye movements as an exception, presumably because the link between our eyes and spatial attention is closer than for other effectors. Another important aspect to consider is that we currently do not know how the grip force data reflect overt and covert shifts of spatial attention. We assume that this relationship is simply symmetric and that attentional shift to the left or right side will lead to increasing grip forces in the left or right hand, respectively. Yet, at the moment no studies have validated this simple intuition.

ATOM predicts either an increase of force with number magnitude, as Krause et al. (2013) demonstrated, or else a decrease if participants associate larger numbers with a wider grip (Andres et al., 2004; see *Hypothesis 2* in our “Introduction” section). In the present study, no automatic mapping between applied force and numbers (FoNA) was found at the group level, neither continuous nor categorical. However, at the individual level, one participant did demonstrate significant FoNA, and the majority of participants tended to associate a wider grip aperture with larger number magnitudes. The weakness of these effects and their high inter-individual variability deserves an explanation: the FoNA discovered previously was only categorical (Fischer and Miller, 2008; Vierck and Kiesel, 2010), and was only studied in experiments where force was a factor discriminating responses and was given in the instruction, i.e., the same paradigm that is described by the dual-route model for the SNA effect. The mapping between grip aperture and numerical magnitude also either explicitly included the dimension of interest in the instruction (Andres et al., 2004; Ranzini et al., 2011), thus making it a part of a conditional route, or used objects of different sizes (Lindemann et al., 2007; Andres et al., 2008; Namdar et al., 2014) which might draw participants’ attention to this feature and make it conscious.

Taken together with the results of the present study, these observations allow us to specify ATOM in the motor domain more precisely: (1) magnitude mapping is flexible and context- (task-, setup-, stimuli-) specific; (2) this mapping does not always happen automatically but requires either a conditional route including the dimension of interest (grip aperture or force production), or implicit variability in the performed action, which suggests participants dimension of mapping. To the best of our knowledge, no studies have yet demonstrated a continuous effect of numerical magnitude on the force, and only the categorical pattern was found, unlike the SNA effect in a parity judgment task. It might mean that qualitatively different mechanisms may underlie SNA and FoNA.

Although no systematic SNA or FoNA were found at the group level (*Hypothesis 4*), it could still be the case that different participants rely on different representations (inter-individual variability, *Hypothesis 5*), some on space- and others on ATOM-related, and by aggregating the data together we diminish both effects. However, analysis at the individual level excludes this possibility: only one participant demonstrated reliable SNA, and another one demonstrated FoNA. Moreover, no negative correlation between the two effects was found. If SNA and FoNA reflect inter-individual differences, then their strengths should correlate negatively: Participants, who rely on spatial representations, should have weaker non-spatial representations and* vice versa*. Instead, in the original study by Krause et al. (2013), a non-significant, but *positive* correlation between the strength of the two effects was found. It is also known that in the parity judgment task the SNA effect describes a linear relationship between number magnitude and left/right space (Wood et al., 2008), while the relationship between magnitude and force was only categorical in previous studies, as reviewed above. This categorical relationship is more similar to the SNA observed in a magnitude comparison task. This consideration also points to qualitatively different processes underlying the two measures.

## Conclusion

A unified model of number cognition, that integrates the existing accounts of magnitude representations and that predicts the involvement of different types of number representations is still missing. The present research examined three accounts for magnitude representations: MNL, A Theory Of Magnitude (ATOM), and Embodied Cognition. Each of these accounts is supported by empirical data, but they all lead to different predictions. The present study explicitly tested all three models by employing a novel method in numerical cognition—grip force registration. The obvious advantages of this method are its high temporal resolution and the possibility to use a no-go paradigm, excluding any explicit motor response.

Force fluctuations were found to reflect stages of mental processing in both specific (i.e., motor system-related) and nonspecific ways (i.e., general cognitive processes-related, such as initial stimulus processing, decision making, or memory). Both a significant effect of finger counting preference (100–140 ms) and an effect of number magnitude (410–450 ms) on spontaneous grip force fluctuation were found, thus indicating two steps of numerical processing (Hsu and Szücs, 2012). An effect of finger counting was also found at the stage of encoding the stimulus into working memory (570–600 ms). No clear automatic number-space (MNL) or number-force (ATOM) associations were revealed at the group level, although some evidence supporting inter-individual variations of ATOM-based representations was observed: While one participant in the sample demonstrated significant direct automatic mapping of number magnitude on force (as in Krause et al., 2013), the majority of participants tended to map number magnitude on grip width (as in Andres et al., 2004), with larger numbers corresponding to wider grips and thus weaker grip forces.

There are fundamental differences between the three accounts of numerical cognition: The SNA effect is strongly influenced by cultural factors, specifically by participants’ reading direction (Shaki et al., 2009), it is highly flexible (Bächtold et al., 1998; Fischer et al., 2010) and it increases with age (Wood et al., 2008). Altogether, this indicates that SNA is rather a sign of a cultural, i.e., learned mechanism of symbolic substitution (but see Shaki and Fischer, 2018a). On the other hand, Walsh (2015) argues that the generalized magnitude system encompassed by ATOM has inborn physiological underpinnings: “more” in one dimension simply matches “more” in another dimension (see prothetic and metathetic dimension: Stevens and Galanter, 1957; Lindemann and Fischer, 2015). Finger counting is a combination of both: more fingers mean more objects; at the same time a spatial mapping is introduced. Perhaps this intermediate role of finger counting—being a combination of an intuitively accessible mapping and a learned symbolic substitution—makes this mechanism such an efficient and universal tool for learning abstract numerical concepts.

Although our study only includes a small sample and is rather exploratory, it introduces a new and potentially highly informative method to study various aspects of numerical cognition: detailed timing of processing, nature of mental representations, and individual differences in number processing. The technique of bimanual grip force recording is easy in implementation and it suggests straightforward interpretations. We hope that its use will advance the field of numerical cognition research.

## Data Availability Statement

The datasets generated for this study can be found in the Open Science Framework (OSF) at https://osf.io/apzt7/ (doi: 10.17605/OSF.IO/APZT7).

## Ethics Statement

The studies involving human participants were reviewed and approved by Ethics Committee, University of Potsdam. The patients/participants provided their written informed consent to participate in this study.

## Author Contributions

AM, OL, and MF contributed to the design and implementation of the research, to the analysis of the results, and the writing of the manuscript. All authors contributed to the article and approved the submitted version.

## Conflict of Interest

The authors declare that the research was conducted in the absence of any commercial or financial relationships that could be construed as a potential conflict of interest.

## Footnotes

^1^Hereafter, people who start counting on their left vs. right hand are referred to as left-starters and right-starters, respectively.

^2^In this section, continuous means that the effect is gradually increasing/decreasing according to the number magnitude, e.g., 1 is associated with the left side stronger than 2; 2—stronger than 3 etc. Instead, a categorical effect means that the number magnitude is modelled as being split into two intervals: small (from 1 to 4) and large (from 6 to 9), without any differences between numbers within each of the intervals.

^3^Their method requires a 200 ms subtraction for comparability: Auditorily presented words are discriminated at the “uniqueness point” where they cannot be confused with any other existing word in the language. For words in a context, this moment appears approximately 200 ms after word onset (Marslen-Wilson, 1984). In Myachykov et al. (2016) only four words were presented (one, three, seven, nine), so it is plausible that discrimination occurred around 200 ms after stimulus onset.

^4^Calculated by using the formula 57.3 * w/d (w—width of the object; d—distance to the object).

^5^Data about musical training were collected because, while learning some instruments (e.g., guitar or piano), participants might have acquired new finger-number associations that are required in such training. We are not aware of any studies demonstrating systematic influence of such associations on number processing, but decided to control for this factor given our hand-held recording apparatus.

^6^z′ = 0.5*[ln(1 + r) − ln(1 − r)]

^7^A larger range (± 500 mN) compared to Nazir et al. (2017) (± 200 mN) was chosen because longer epochs were selected (1,200 instead of 1,000 ms). Obviously, the longer the epoch, the higher the probability that force will exceed a certain threshold because of increased variability. This would lead to the loss of data. Thus, wider thresholds should be used for longer trials/epochs.

^8^ANOVAs were performed in STATISTICA 8.0 (StatSoft, Inc., 2007)

^9^Note that our sample size is rather small (*N* = 26) compared to standard samples in individual differences research. Thus, the analysis in this section should be considered as an exploratory one. However, we hope that the research model suggested here can be useful for future studies on the topic.

^10^See P300 (Gevins and Cutillo, 1993) and no-go P3 in EEG recordings (Verleger et al., 2016).
